# Antibody Mediated Rejection is not Associated with Worse Survival in Adherent Heart Transplant Patients in the Contemporary Era

**DOI:** 10.1101/2023.12.01.23299311

**Published:** 2023-12-08

**Authors:** Paul J. Kim, Vincenzo Cusi, Ashley Cardenas, Yuko Tada, Florin Vaida, Nicholas Wettersten, Jennifer Chak, Priyesha Bijlani, Victor Pretorius, Marcus Anthony Urey, Gerald P. Morris, Grace Lin

**Affiliations:** 1 UC San Diego Health, San Diego, CA.; 2 Department of Pathology, University of California, San Diego, California, USA; 3 Department of Family Medicine and Public Health, UC San Diego, La Jolla, CA.; 4 Cardiology Section, Veterans Affairs San Diego Healthcare System, San Diego, CA.; 5 Division of Cardiovascular and Thoracic Surgery, Department of Surgery, University of California, San Diego, California, USA

**Keywords:** antibody mediated rejection, Complement C4, donor specific antibodies, heart transplantation, cardiac allograft dysfunction, C4d, biopsy-negative rejection, nonadherence

## Abstract

**Background:**

C4d immunostaining of surveillance endomyocardial biopsies (EMB) and testing for donor specific antibodies (DSA) are routinely performed in the first year of heart transplantation (HTx) in adult patients. C4d and DSA positivity have not been evaluated together with respect to clinical outcomes in the contemporary era (2010–current).

**Methods:**

This was a single center, retrospective study of consecutive EMBs performed between November 2010 and April 2023. The primary objective was to determine whether history of C4d and/or DSA positivity could predict death, cardiac death, or retransplant. Secondary analyses included cardiac allograft dysfunction and cardiac allograft vasculopathy. Cox proportional hazards models were used for single predictor and multipredictor analyses.

**Results:**

A total of 6,033 EMBs from 519 HTx patients were reviewed for the study. There was no significant difference (p = 0.110) in all-cause mortality or cardiac retransplant between four groups: C4d+/DSA+, C4d+/DSA−, C4d−/DSA+, and C4d−/DSA−. The risk for cardiac mortality or retransplant was significantly higher in C4d+/DSA+ versus C4d−/DSA− patients (HR = 4.73; p_c_ = 0.042) but not significantly different in C4d+/DSA− versus C4d−/DSA− patients (p_c_ = 1.000). Similarly, the risk for cardiac allograft dysfunction was significantly higher in C4d+/DSA+ versus C4d−/DSA− patients (HR 3.26; p_c_ = 0.001) but not significantly different in C4d+/DSA− versus C4d−/DSA− patients (p_c_ = 1.000). Accounting for nonadherence, C4d/DSA status continued to predict cardiac allograft dysfunction but no longer predicted cardiac death or retransplant.

**Conclusions:**

Medically adherent C4d+/DSA+ HTx patients show significantly greater risk for cardiac allograft dysfunction but not cardiac mortality or retransplant. In contrast, C4d+/DSA− patients represent a new immunopathologic group with a clinical course similar to that of HTx patients without antibody mediated rejection.

## Introduction

The pathologic criteria for antibody mediated rejection (AMR) in heart transplant (HTx) patients have evolved since their early description,^1^ to the current application of immunopathologic criteria that includes histopathologic findings such as endothelial swelling and intravascular macrophages (marked by immunohistochemical staining for CD68) and/or evidence of antibody mediated pathology through immunostaining of complement proteins C3d or C4d, as defined by the International Society for Heart and Lung Transplantation (ISHLT) Working Formulation in 2013.^2,3^ Subsequently, C4d immunostaining has become the most commonly described method for pathologic antibody mediated rejection (pAMR) grading.^4–9^

As a result, the immunopathologic criteria from the ISHLT created five different pAMR grades, including two subtypes of pAMR1 – pAMR1(I^+^) and pAMR1(H^+^). However, the clinical significance of C4d positivity itself remains an area of active research, especially since the 2013 ISHLT Working Formulation and 2015 American Heart Association Scientific Statement.^2–4,6,7,9–11^ In addition, the association of donor specific antibody (DSA) positivity and poor clinical outcomes has been previously reported.^5,12^ However, additional literature have reported conflicting results for the association of positive DSA and clinical outcomes based on anti-HLA (human leukocyte antigen) class type.^13–15^ Thus, this study evaluated the clinical significance associated with C4d and/or DSA positivity in adult HTx patients with respect to clinical outcomes in the contemporary era (2010–current).

## Methods

### Data Sharing

Data, methods, and materials used to conduct the research are available from the corresponding author upon reasonable request.

### Study Design

Consecutive heart transplant (HTx) patients who were 18 years of age or older and underwent right ventricular endomyocardial biopsy (EMB) between November 2010 to April 2023 were retrospectively reviewed. The typical EMB surveillance protocol at the University of California, San Diego Health (UC San Diego Health) has been described previously.^16^ This protocol includes C4d immunofluorescence at 2 and 4 weeks, and at 3, 6 and 12 months during surveillance EMBs in addition to for-cause EMB indication.^2,3^ DSA testing is also performed at the same time intervals for surveillance indication and whenever there is clinical concern for AMR. The authors (VC, AC, PB, JC) collected patient data and clinical outcomes from the electronic medical record. Approval for this study was provided by the UC San Diego Health Office of IRB Administration (IRB #805675). This study adheres to the principles of the Declaration of Helsinki formulated by the World Medical Association and the US Federal Policy for the Protection of Human Subjects.

### Inclusion and Exclusion Criteria

HTx patients with C4d and DSA testing performed were included for this study. Patients diagnosed with pAMR without positive C4d immunofluorescence (i.e., by histology or CD68 positivity)^2^ were excluded from the study.

### Pathologic Tissue Exams and Anti-HLA Antibody Testing

The results of pathologic tissue exams were collected for each EMB. C4d immunofluorescence was performed starting November 2010 and positivity was defined according to the ISHLT Working Formulation.^2^ Acute cellular rejection (ACR)^17^ and pAMR grading^2^ were performed according to ISHLT guidelines. All patients were tested for anti-HLA antibodies as standard of care treatment. Patients were tested post-HTx for anti-HLA antibodies as previously described.^18^ DSA were identified by comparison of antibody testing results to donor HLA typing. Normalized mean fluorescence intensity values > 3,000 were used to identify positive alloantibodies.

### Clinical Outcomes and Variables

All patients were followed for all-cause death. Cause of death was adjudicated by two experienced HTx cardiologists (NW and PJK). When there was a disagreement, a third cardiologist (YT) made the final determination. Other clinical outcomes included: ISHLT cardiac allograft vasculopathy (CAV) grade 2 or greater^19^, cardiac retransplant, cardiac allograft dysfunction (echocardiogram demonstrating LVEF < 50% and excluding primary graft dysfunction^20^), and future episodes of AMR and/or ACR. Immunomodulatory treatment for AMR refers to a significant change in a subject’s immunomodulatory regimen as described previously.^21^

### Medical Nonadherence

Documentation by any clinical team member of medical nonadherence after HTx was recorded by the authors (VC, AC, PB, JC). Testing results for illicit substances in nonadherent C4d+ patients who subsequently died was also recorded, if available.

### Statistical Analysis

Continuous variables were expressed as mean + standard deviation (SD) for normally distributed variables or median and interquartile range (IQR) for non-normally distributed variables and compared with the use of the Student’s t-test or Wilcoxon rank-sum test, respectively. The Kruskal-Wallis test was used for multiple group comparisons of continuous data. Categorical variables were expressed as counts and percentages and compared between groups using either the Pearson’s chi-square or Fisher’s exact test whenever any observed counts were ≤ 5. If the null hypothesis was rejected, pairwise group comparisons using the Bonferroni-Holm procedure were subsequently performed. The agreement rate between the two adjudicators for clinical outcomes was performed using Cohen’s kappa statistics.

The association of C4d/DSA groups with time to event outcomes was evaluated using single predictor and multipredictor Cox proportional hazards models. The multipredictor model adjusted for potential confounders (recipient, donor, and transplant characteristics) via backward model selection with p-value ≤ 0.15 threshold for inclusion. Additional exploratory analyses investigated factors associated with time to event outcomes using Cox models applying a forward model selection procedure. Medical nonadherence was only evaluated as a potential predictor in exploratory analyses. Cause-specific hazard models were used to address etiologic questions. The Fine-Gray subdistribution hazard models were performed to evaluate the effect of covariates on competing risks as sensitivity analyses. Collinearity was evaluated by calculating the variance inflation factor for each independent variable.

Analysis was conducted in R (R Core Team, 2022) with survival (v3.5–5)^22^ for Cox proportional hazards regression, cmprsk (v2.2–11) for Fine-Gray subdistribution hazard models^23^, fmsb (v0.7.5) for Cohen’s kappa,^24^ and car (v3.1–2) for variance inflation factor.^25^ Figures were produced using the package ggplot2.^26^ The corrected p-values are designated as p_c_. For single hypothesis testing we report the uncorrected p-value unless stated otherwise. A p or p_c_ < 0.05 are considered significant.

## Results

### Patient Demographics

**Figure 1.**
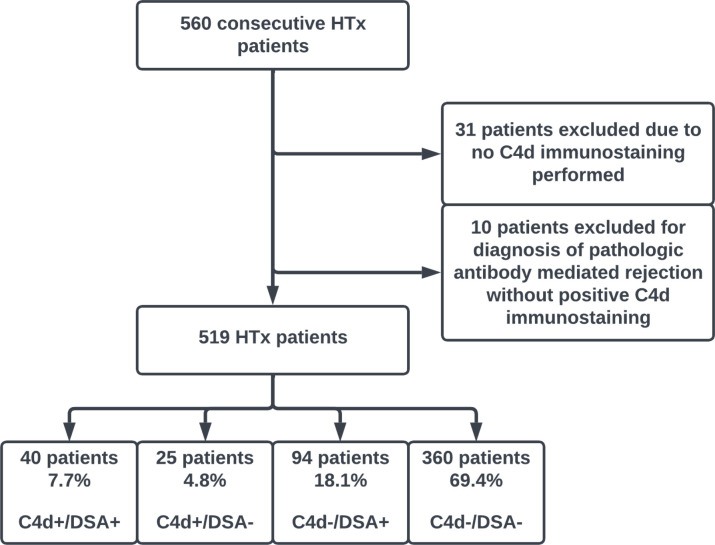
Flow diagram. HTx, heart transplantation; DSA, donor-specific antibodies.

A total of 560 HTx patients were identified and deemed potentially eligible for the study (Figure 1). Of these, 31 patients were excluded for not having C4d immunofluorescence performed. As our study was specific to C4d and DSA positivity, 10 patients were also excluded for the diagnosis of pAMR using CD68^+^ immunostaining or histology criteria without C4d positivity.^2^

Baseline characteristics of the study population are depicted in Table 1. Patients were typically male (81%) and non-Hispanic white (41%) with a mean age of 54 ± 14 years at the time of HTx. HTx recipients were followed for a total of 1,855.1 person-years in this analysis from time of HTx to end of follow-up.

The primary reason for transplantation was non-ischemic cardiomyopathy (60%). Highly sensitized (panel of reactive antibodies, PRA >= 10%) patients comprised 19% of the study cohort. Induction therapy was used in 246 patients (47%) and 73 patients (13%) underwent multi-organ transplants. There were 89 patients (16%) that had a documented history of medical nonadherence post-HTx. Significant group differences were seen for recipient age (p < 0.001) and a trend for differences observed with respect to recipient female sex (p = 0.070) and allosensitization pre-HTx (p = 0.067).

### C4d and DSA positivity

**Figure 2.**
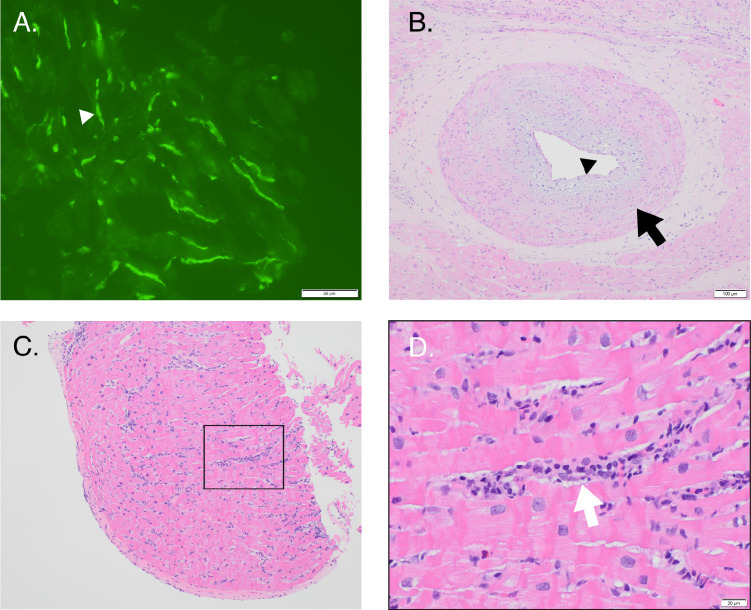
Representative photomicrographs of a C4d+/DSA+ patient. A. Strong, diffuse endothelial staining (white arrowhead) of capillaries by C4d immunofluorescence (40x). B. Cardiac allograft vasculopathy with concentric intimal fibrosis (black arrow) and endothelial swelling (black arrowhead). C and D. Hematoxylin and eosin staining at 10x (C) and 40x (D) demonstrating intramuscular distribution of macrophages (white arrow) with associated endothelial swelling. DSA, donor-specific antibodies.

A total of 6,033 EMBs from 519 patients, including 4 cardiac retransplants, were evaluated. Patients were divided into 4 groups based on history of C4d and DSA positivity. There were 40 (7.7%) C4d+/DSA+, 25 (4.8%) C4d+/DSA−, 94 (18.1%) C4d−/DSA+, and 360 (69.4%) C4d−/DSA− patients (Figure 2). There were 65 (12.5%) patients that had C4d positivity on immunofluorescence and 134 (25.8%) patients with DSA positivity. Of the positive DSAs, 122 (94.6%) were de novo, 55 (41.0%) were class 1 DSAs, 109 (81.3%) were class 2 DSAs, and 31 (23.1%) were both class 1 and 2 DSAs. Most C4d+ EMBs (92.3%) occurred in the setting of ACR grades 0R or 1R. CAV was known or diagnosed at the time of C4d positivity in 5 of the 20 patients that had been evaluated for CAV at the time. Cardiac allograft dysfunction was known or diagnosed at the time of C4d positivity in 16 (24.6%) patients.

### Association of C4d and DSA status with all cause death, cardiac death, or retransplant

Of the 519 patients, 58 (11.2%) died or underwent cardiac retransplant during the follow-up period (Supplementary Table S1). Of the deaths, 5 (9.3%) were due to cancer, 16 (29.6%) were due to cardiac causes, 22 (40.7%) were due to infection, 7 (13.0%) were due to other causes, and 4 (7.4%) were due to unknown causes. Initial adjudication of cause of death was in agreement 88.1% of the time with a Cohen’s kappa of 0.84 (0.72, 0.96; p < 0.001).

**Figure 3.**
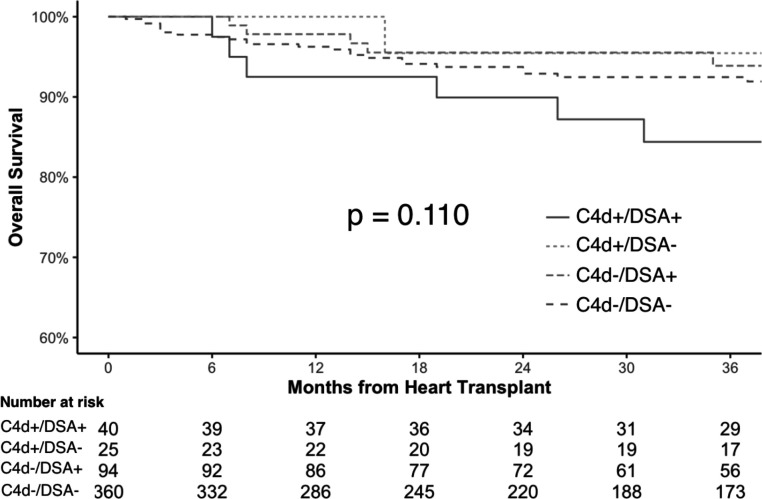
Kaplan-Meier Curves for Overall Survival by C4d/DSA status. There was no significant difference in overall survival between groups by C4d/DSA status (p = 0.110). DSA, donor-specific antibodies.

**Figure 4.**
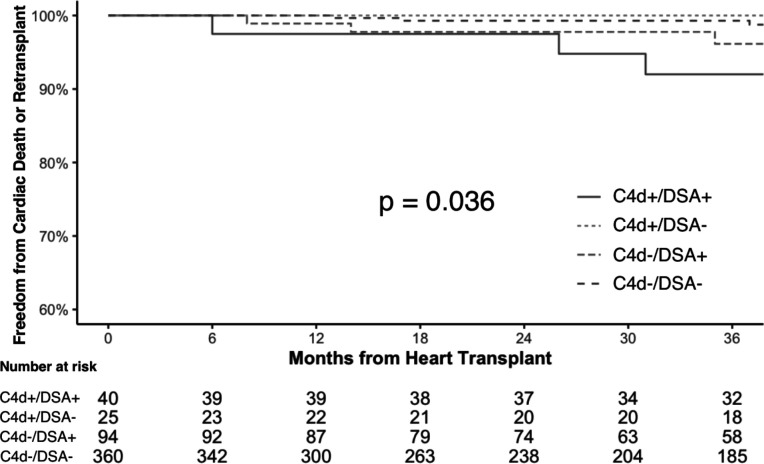
Kaplan-Meier Curves for Freedom from Cardiac Death or Retransplant. There was significantly higher cardiac mortality or retransplant in C4d+/DSA+ compared to C4d−/DSA− patients (p_c_ = 0.042). However, there was no significant difference in cardiac mortality or retransplant in C4d+/DSA− compared to C4d−/DSA− patients (p_c_ = 1.000). DSA, donor-specific antibodies.

There was no significant difference in all-cause mortality or cardiac retransplant between the four C4d/DSA groups (Figure 3; p = 0.110). However, there was a significantly higher risk in cardiac mortality or retransplant in C4d+/DSA+ compared to C4d−/DSA− patients (Figure 4; hazard ratio [HR] = 4.73; 95% confidence interval [CI] 1.57–14.27; p_c_ = 0.042). There was no significant difference in cardiac mortality or transplant in C4d+/DSA− compared to C4d−/DSA− patients (p_c_ = 1.000). The Fine-Gray subdistribution hazard model also demonstrated consistent findings with the cause-specific hazard model (p = 0.001). Of note, we observed all cardiac deaths of patients with C4d positivity occurred in-hospital.

### Predictors for clinical outcomes

For all-cause mortality or cardiac retransplant, we found cardiac allograft dysfunction, medical nonadherence, and female donor to male recipient sex mismatch to be significant independent risk factors (Supplementary Table S2). For cardiac mortality or retransplant, cardiac allograft dysfunction, medical nonadherence, CAV, and percutaneous mechanical circulatory support post-HTx (Table 2 and Supplementary Table S3) were found to be significant independent predictors.

For prediction of DSA positivity, we found recipient age, medical nonadherence, and allosensitization status pre-HTx by PRA to be significant independent risk factors (Supplementary Table S4). For prediction of C4d positivity, we found class 1 and 2 antibodies, medical nonadherence, and female recipient sex to be significant independent predictors (Supplementary Table S5).

For cardiac allograft dysfunction, we found C4d/DSA status, medical nonadherence, CAV, and donor body mass index to be significant independent predictors (Table 3, Supplementary Tables S6 and S7). Post hoc analysis of C4d/DSA status demonstrated significantly higher risk for cardiac allograft dysfunction in C4d+/DSA+ versus C4d−/DSA− patients (HR 3.16; 95% CI, 1.51–6.61; p_c_ = 0.013). There was no significant difference in risk for cardiac allograft dysfunction in C4d+/DSA− (p_c_ = 1.000) and C4d−/DSA+ (p_c_ = 1.000) versus C4d−/DSA− patients. We did not find the severity of ACR grade with concurrent C4d positivity correlated with cardiac allograft dysfunction (p = 0.227). We also did not find the severity of pAMR grade with concurrent C4d positivity (p = 0.313) nor the number of AMR episodes (p = 0.742) correlated with cardiac allograft dysfunction.

With respect to future CAV, the majority of HTx patients were evaluated for CAV by coronary angiography including: 360 (69.4%) of the original patient cohort, 54 (83.1%) of the C4d+ patients, 19 (82.6%) of the C4d+ patients with cardiac allograft dysfunction, and 12 (85.7%) of the nonadherent C4d+ patients with cardiac allograft dysfunction. Of the 7 nonadherent C4d+/DSA+ patients with cardiac mortality or retransplant, 6 patients had a coronary angiogram performed within 6 months prior to the cardiac death. Given the relatively low number of future CAV events (n = 30), we performed a post hoc pairwise comparisons of the primary variable of interest, C4d/DSA patient groups, and adjusted for identified confounders (Supplementary Table S8). We observed the point estimate for C4d+/DSA+ versus C4d−/DSA− patients was associated with a higher risk of CAV, though this was not statistically significant after correcting for multiple comparisons (HR 3.52; 95% CI, 1.21–10.24; p_c_ = 0.063). The C4d+/DSA− patients (p_c_ = 0.527) did not have a significantly higher risk for CAV versus C4d−/DSA− patients.

### Comparison of C4d/DSA groups

We found no significant difference in cardiac index by right heart catheterization in C4d+/DSA+ (2.6 ± 0.8 LPM/m^2^) compared to C4d+/DSA− patients (2.8 ± 0.6 LPM/m^2^; p = 0.182) at the time of C4d positivity. However, median pulmonary capillary wedge pressure was significantly higher in C4d+/DSA+ (17 mmHg; IQR, 12–22 mmHg) compared to C4d+/DSA− patients (13 mmHg; IQR, 8–16 mmHg; p = 0.015).

Treatment for AMR varied widely and was significantly more likely in C4d+/DSA+ compared to C4d+/DSA− patients (80.0% vs 24.0%; OR 12.03; 95% CI, 3.34–50.73; p < 0.001). Intravenous immunoglobulin (62.5% vs 16.7%, p = 0.071) and plasmapheresis (59.4% vs 16.7%, p = 0.082) showed a trend of being given more often to treated C4d+/DSA+ compared to C4d+/DSA− patients. In contrast, we found no significant difference in use of intravenous methylprednisolone (43.8% vs 50%, p = 1.0), oral prednisone 40 mg/day or higher (21.9% vs 33.3%, p = 0.613), antithymocyte globulin (15.6% vs 0, p = 0.570), or rituximab (12.5% vs 16.7%, p = 1.0) in treated C4d+/DSA+ compared to C4d+/DSA− patients.

Separate episodes of future treated AMR occurred in 25% of C4d+/DSA+ patients. C4d+/DSA+ patients showed a trend towards greater proportion of future treated ACR episodes compared to C4d−/DSA− patients (OR 2.54; 95% CI, 0.98–6.04; p_c_ = 0.066), while C4d+/DSA− patients did not show a significant difference compared to C4d−/DSA− patients (p_c_ = 0.512). There was no significant difference in proportion of mixed rejection between the C4d+/DSA+ and C4d+/DSA− groups (7.5% vs 8.0%; p = 1.000). Of note, all C4d+/DSA− patients eventually became C4d negative with a median of 3.1 weeks (IQR, 2.1–4.6 weeks).

The C4d+/DSA+ group showed a significantly greater median time to develop C4d positivity after HTx compared to the C4d+/DSA− group (Supplementary Figure S1; 33.6 vs 3.6 weeks; p = 0.004). For the C4d+/DSA+ group, 36 (90.0%) patients demonstrated positive DSA concurrent with C4d positivity and the median time from DSA to C4d positivity was 4.5 days (IQR, 0.0–41.0 days) for these patients. Of the 4 patients that did not demonstrate positive DSA concurrent with C4d positivity, 3 patients demonstrated positive DSA within 1 year of C4d positivity while 1 patient demonstrated positive DSA after 1 year. Despite an initially negative DSA, three of the four C4d+/DSA+ patients were still treated for AMR.

Lastly, the C4d+/DSA+ group showed no significant difference compared to the C4d−/DSA+ group with respect to median time to DSA positivity after HTx (24.6 vs 18.3 weeks; p = 0.975). However, the C4d+/DSA+ group showed significantly greater concurrent class 1 and 2 DSAs compared to the C4d−/DSA+ group (OR 7.40; 95% CI, 2.86–20.18; p < 0.001).

### Medical nonadherence by C4d/DSA status

**Figure 5.**
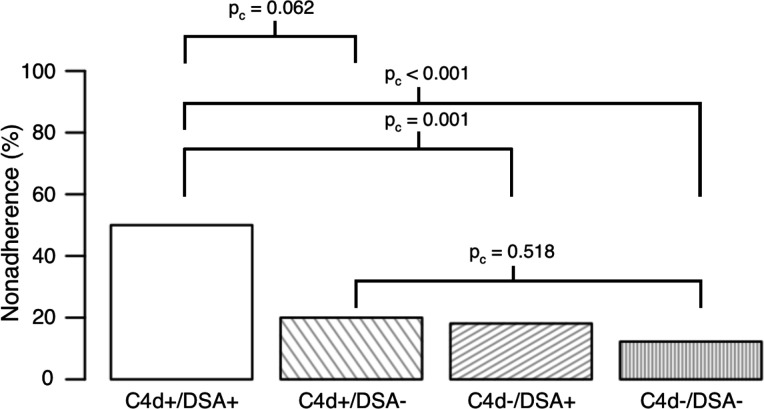
Proportion of Medical Nonadherence by C4d/DSA Status. C4d+/DSA+ group showed a significantly increased proportion of patients with medical nonadherence compared to both C4d− groups. However, C4d+/DSA− patients did not show a significant difference compared to C4d−/DSA− patients. DSA, donor-specific antibodies.

We observed a significant difference in medical nonadherence by C4d/DSA status (Figure 5; p < 0.001). In pairwise comparisons, medical nonadherence was significantly higher in C4d+/DSA+ compared to C4d−/DSA− patients (OR 7.13; 95% CI, 3.35–15.22; p_c_ < 0.001). In comparison, C4d+/DSA− patients did not show a significant difference compared to C4d−/DSA− patients (OR 1.79; 95% CI, 0.50–5.26; p_c_ = 0.518). We also found nonadherent patients were significantly less likely to be at their target immunosuppressive drug trough levels at the time of C4d positivity (61% vs 92%; OR 0.13; 95% CI, 0.02–0.64; p = 0.006). Of note, testing for illicit substances was performed by the clinical team in 8 (61.5%) nonadherent C4d+ patients who had an outcome of death. One patient (12.5%) was positive for methamphetamine, one patient (12.5%) was positive for unprescribed benzodiazepines, and one patient (12.5%) was positive for unprescribed opiates.

## Discussion

In this retrospective cohort of 519 HTx patients with greater than 1,800 patient-years of follow-up, we observed the following key findings. First, C4d+/DSA+ patients demonstrated significantly higher cardiac morbidity, mortality, or retransplant when compared to C4d+/DSA− patients. Second, C4d+/DSA+ patients demonstrated significantly higher medical nonadherence compared to C4d+/DSA− patients. Third, after accounting for nonadherence, C4d+/DSA+ status still predicted higher cardiac allograft dysfunction but no longer predicted higher cardiac mortality or retransplant. Overall, these findings help to understand the clinical significance of DSA positivity in the setting of C4d positivity in HTx patients.

AMR is typically diagnosed by pathologic criteria, set forth in the last Working Formulation by the ISHLT, which has greatly improved the sensitivity for the diagnosis of pAMR.^2,27^ However, we show that the new immunopathologic criteria and the recommendation to routinely perform DSA testing^28^ in the first year after HTx contributed to the classification of a C4d+/DSA− group that has not been well described in the contemporary era.^5,9,15^ In the current study, we find that C4d+/DSA− patients are a clinically distinct group compared to C4d+/DSA+ patients. When accounting for medical nonadherence, C4d+/DSA+ patients demonstrated a significantly higher risk in cardiac allograft dysfunction and a trend towards higher CAV incidence compared to C4d+/DSA− patients. Recently, RNA-sequencing of EMBs also demonstrated transcriptomic differences between DSA+ and DSA− groups in AMR patients, with significant upregulation of genes related to immunity in DSA+ AMR patients.^10^ Thus, we suggest that C4d+/DSA− patients represent a new immunopathologic group, both clinically and transcriptomically.

The onset of C4d positivity occurred significantly earlier in the C4d+/DSA− group, at 1 month post-HTx, compared to 9 months post-HTx in the C4d+/DSA+ group. Although initial AMR literature described early onset of positive cardiac immunostaining,^29,30^ more recent literature describe a bimodal distribution of “early” and “late” C4d immunostaining.^4,5,8,31–34^ A potential reason for early positive C4d immunostaining has been previously attributed to complement activation related to reperfusion injury at the time of HTx.^35–37^ Non-HLA antibodies are another possibility for early positive C4d immunostaining.^38^ Our study findings support the hypothesis that the mechanism for complement activation and deposition in C4d+/DSA− patients is different from C4d+/DSA+ patients and deserves further study. Other immunopathological biomarkers including phosphorylated p70 S6 Kinase and S6 ribosomal protein also may help differentiate the C4d+/DSA+ and C4d+/DSA− groups.^39^

We also found the clinical differences at time of presentation to be significantly different for the C4d+/DSA+ compared to C4d+/DSA− patients. The majority (98%) of C4d+/DSA+ patients demonstrated DSA positivity either concurrent with or within a year of C4d positivity. Additionally, C4d+/DSA+ patients presented with significantly higher intracardiac filling pressures^4,40^ at the time of C4d positivity compared to C4d+/DSA− patients. Consequently, the treatment of the minority of C4d+ patients without concurrent DSA positivity did not appear to be different compared to C4d+ patients with concurrent DSA positivity. While the majority of C4d+/DSA+ patients received some type of immunomodulatory treatment in addition to their baseline immunosuppression regimen, the immunomodulatory treatments varied widely, with intravenous immunoglobulins and plasmapheresis being more frequently used compared to others.^3,4^

A novel finding from our study is that medical nonadherence is significantly correlated to C4d+/DSA+ status and ultimately, cardiac death or retransplant. We found medical nonadherence to be significantly associated with DSA positivity, C4d positivity, cardiac allograft dysfunction, and cardiac death or retransplant. However, we did not find medical nonadherence to be significantly associated with CAV. We believe this finding does not contradict prior literature describing correlation observed between AMR and CAV.^4,6,15,33,40–42^ Instead, our study results complement current literature and suggest nonadherence contributes to mortality through an independent mechanism(s). An important and related observation is that while adherent C4d+/DSA+ patients are at a higher risk for cardiac allograft dysfunction, they do not demonstrate a higher risk for cardiac death or retransplant as do nonadherent C4d+/DSA+ patients. While C4d+/DSA+ status predicted cardiac allograft dysfunction, it did not predict cardiac death or retransplant, suggesting other factors including nonadherence were more significant contributors. Of note, we did not find a correlation with cardiac allograft dysfunction and severity of pAMR grading, number of AMR episodes, nor mixed acute rejection. Further investigation of cardiac allograft dysfunction and its mechanistic relationship to cardiac death or retransplant will be necessary to better understand and treat AMR.

In our study cohort, class 2 DSA positivity was a relatively common occurrence (21%),^5,8,14,15,43^ while HTx patients with both class 1 and 2 DSA positivity was much less frequent (6%). Furthermore, we found that patients who were both class 1 and 2 DSA positive were at a significantly higher risk for being in the C4d+/DSA+ rather than C4d−/DSA+ group. This observation contributes to existing literature^5,15^ and is important for HTx patients that become DSA positive as we did not find the majority of these patients (i.e., C4d−/DSA+ group) to be at a higher risk for all cause death, cardiac death or retransplant, CAV, and cardiac allograft dysfunction compared to C4d−/DSA− patients. Further study of class 1 and 2 DSAs, including at a higher molecular resolution,^18,44^ may provide additional insights of the risks specific DSAs pose to HTx patients.

Because both the majority of C4d+/DSA+ patients received some form of immunomodulatory treatment and the therapies varied widely, we could not make any confident conclusions on the effect of treatment on clinical outcomes within the C4d+/DSA+ group. Randomized control trials to identify C4d+/DSA+ patients that would benefit from treatment and also specify the optimal type of immunomodulatory treatment will be critical going forward.^4,21,45,46^

Finally, our study adds to the evolving literature of “biopsy-negative rejection” after HTx.^47^ We show cardiac allograft dysfunction occurs most frequently in the C4d+/DSA+ group but also occurs in other C4d− groups without an identified cause. With continued research in non-HLA antibodies^38^ and immunopathology, particularly with RNA-sequencing,^10,48,49^ we believe we will see further classification of immunopathologic phenotypes and greater understanding biologically as well as clinical significance for these patients.

### Limitations

This study should be interpreted within the context of several important limitations. First, this was a retrospective study from a single center and may not necessarily represent the experience of other centers with different patient demographics and variations in post-HTx management. Second, the number of cardiac deaths or retransplants were low for all groups. However, we still found the time to cardiac death or retransplant to be significantly different in C4d+/DSA+ compared to C4d−/DSA− patients but this was no longer significant after accounting for medical nonadherence. Third, while our study represents a sizable cohort relative to other studies,^4–6,13,30^ the C4d+/DSA− group represented a minority of total HTx patients. Thus, our study remains underpowered to detect smaller differences and larger, multicenter studies should be performed to confirm our findings. Fourth, we chose C4d immunofluorescence as a prognostic biomarker^9^ instead of pAMR grading because its simplicity and potential concerns of “overcalls” of pAMR2 without C4d positivity.^50^ However, we recognize there is ongoing research of pAMR classifications and more study in this area is needed.^10,48^

## Conclusions

C4d immunostaining and DSA remain important biomarkers for HTx patients with AMR; however, their significance depends on their clinical context. In medically adherent patients, C4d+/DSA+ status is associated with a higher risk of cardiac allograft dysfunction but not a higher risk of cardiac death or retransplant compared to HTx patients without AMR. Our results may have important future clinical implications by providing clinicians contemporary evidence for the significance of the currently recommended C4d immunostaining and DSA testing in HTx patients.

## Supplementary Material

Supplement 1

## Figures and Tables

**Table 1.** Subject clinical characteristics. BMI, body mass index; CMV, cytomegalovirus; DCD, donation after cardiac death; HTx, heart transplantation; ICM, ischemic cardiomyopathy; MCS, mechanical circulatory support; NICM, nonischemic cardiomyopathy; PHM, predicted heart mass; PRA, panel reactive antibodies

	C4d+/DSA+ Group 1 (n = 40)	C4d+/DSA− Group 2 (n = 25)	C4d−/DSA+ Group 3 (n = 94)	C4d−/DSA− Group 4 (n = 360)	p-value
**Donor characteristics**					
Age, y, mean (SD)	30.97 (9.73)	37.33 (12.23)	31.47 (10.34)	33.08 (10.73)	0.127
Male, N (%)	26 (70.3)	19 (79.2)	74 (79.6)	298 (84.7)	0.118
**Recipient characteristics**					
Age, y, mean (SD)	44.85 (18.70)	59.56 (10.21)	51.19 (15.27)	55.67 (12.86)	<0.001
Male, N (%)	26 (65.0)	20 (80.0)	79 (84.0)	296 (82.2)	0.070
Race					0.232
Asian, N (%)	0	3 (12.0)	6 (6.4)	24 (6.7)	-
Black, N (%)	8 (20.0)	4 (16.0)	14 (14.9)	41 (11.4)	-
Native American, N (%)	0	0	2 (2.1)	2 (0.6)	-
Other Race, N (%)	5 (12.5)	1 (4.0)	11 (11.7)	38 (10.6)	-
Pacific Islander, N (%)	0	1 (4.0)	3 (3.2)	8 (2.2)	-
White, N (%)	27 (67.5)	16 (64.0)	58 (61.7)	247 (68.6)	-
Ethnicity					
Hispanic or Latino, N (%)	14 (35.0)	4 (16.0)	33 (35.1)	105 (29.2)	0.428
Recipient BMI, mean (SD)	26.66 (5.23)	28.47 (5.20)	27.63 (4.82)	26.92 (4.71)	0.280
Indication for Transplant					0.134
NICM, N (%)	25 (62.5)	17 (68.0)	56 (59.6)	210 (58.3)	-
ICM, N (%)	9 (22.5)	4 (16.0)	23 (24.5)	119 (33.1)	-
Mixed ICM/NICM (%)	1 (2.5)	3 (12.0)	6 (6.4)	15 (4.2)	-
Congenital, N (%)	4 (10.0)	0	6 (6.4)	11 (3.1)	-
Cardiac allograft failure, N (%)	1 (2.5)	1 (4.0)	3 (3.2)	5 (1.4)	-
Allosensitization pre-HTx (PRA ≥ 10%), N (%)	10 (32.3)	2 (9.5)	21 (23.9)	51 (16.5)	0.067
Durable MCS, N (%)	11 (27.5)	9 (36.0)	33 (35.1)	131 (36.5)	0.751
**Transplant characteristics**					
Multiorgan transplant, N (%)	6 (15.0)	2 (8.0)	17 (18.1)	49 (13.6)	0.585
Cold ischemic time, min, mean (SD)	198.40 (50.75)	207.70 (60.10)	200.80 (58.66)	199.80 (65.52)	0.693
Sex mismatch (female D-male R), N (%)	3 (8.1)	1 (4.2)	10 (10.8)	28 (8.0)	0.787
PHM difference, % recipient PHM, mean (SD)	6.52 (23.20)	1.40 (20.56)	1.15 (16.80)	5.20 (20.80)	0.400
Induction therapy, N (%)	20 (57.1)	15 (62.5)	45 (48.4)	166 (45.7)	0.358
DCD, N (%)	3 (7.5)	4 (16.0)	12 (12.8)	63 (17.5)	0.338
CMV mismatch (D+/R−), N (%)	6 (15.8)	6 (24.0)	20 (21.5)	72 (20.3)	0.388

**Table 2.** Multipredictor model for cardiac mortality or retransplant as the outcome. AMR, antibody mediated rejection; DCD, donation after cardiac death; HTx, heart transplantation; pMCS, percutaneous mechanical circulatory support.

Variables	HR	95% CI	p-value
Cardiac allograft dysfunction	5.51	[1.87–16.27]	p = 0.002
pMCS post-HTx	5.82	[1.65–20.53]	p = 0.006
Medical nonadherence	3.92	[1.33–11.53]	p = 0.013
Cardiac allograft vasculopathy	3.62	[1.18–11.12]	p = 0.024
Recipient age (per 1-y increment)	0.98	[0.95–1.01]	p = 0.122
DCD	5.56	[0.57–54.67]	p = 0.141

**Table 3.** Multipredictor model for cardiac allograft dysfunction as the outcome. Hazard ratios, confidence intervals, and p-values for pairwise comparisons of the C4d/DSA groups are provided in Supplementary Table S7. BMI, body mass index; DSA, donor-specific antibodies; HTx, heart transplantation; MCS, mechanical circulatory support; PHM, predicted heart mass.

Variables	HR	95% CI	p-value
Donor BMI	0.93	[0.88–0.99]	p = 0.016
Medical nonadherence	2.05	[1.13–3.72]	p = 0.018
Cardiac allograft vasculopathy	2.31	[1.15–4.63]	p = 0.018
C4d/DSA status	-	-	p = 0.032
Durable MCS at time of HTx	1.71	[1.00–2.95]	p = 0.052
PHM difference (per % recipient PHM increment)	1.01	[1.00–1.03]	p = 0.081

## Non-standard Abbreviations and Acronyms

ACR: Acute cellular rejection
AMR: Antibody mediated rejection
CAV: Cardiac allograft vasculopathy
DSA: Donor-specific antibody
ECMO: Extracorporeal membrane oxygenation
EMB: Endomyocardial biopsy
HLA: Human leukocyte antigen
HTx: Heart transplantation
ISHLT: International Society for Heart and Lung Transplantation
LVEF: Left ventricular ejection fraction
pAMR: Pathologic antibody mediated rejection
PRA: Panel of reactive antibodies
PHM: Predicted heart mass
UC San Diego Health: University of California, San Diego Health
