# Supplementary material for: Donor-Specific Antibody Testing is an Effective Surveillance Strategy for High-Risk Antibody Mediated Rejection in Heart Transplant Patients in the Contemporary Era
Source: medRxiv. 2024 Oct 1:2023.12.01.23299311. Preprint. [Version 3] doi: 10.1101/2023.12.01.23299311 (PMC10723500; doi:10.1101/2023.12.01.23299311)
Supplement: 1 [file NIHPP2023.12.01.23299311V3-supplement-1.pdf]

medRxiv preprint doi: https://doi.org/10.1101/2023.12.01.23299311; this version posted October 1, 2024. The copyright holder for this preprint (which was not certified by peer review) is the author/funder, who has granted medRxiv a license to display the preprint in perpetuity.
It is made available under a CC-BY 4.0 International license .

Table S1. Baseline characteristics of heart transplant patients and outcomes for the total study cohort. ACR, acute cellular rejection; AMR, antibody mediated rejection; BMI, body mass index; CMV, cytomegalovirus; DCD, donation after cardiac death; DSA, donor-specific antibodies; HTx, heart transplantation; ICM, ischemic cardiomyopathy; ISHLT, International Society for Heart and Lung Transplantation; MCS, mechanical circulatory support; NICM, nonischemic cardiomyopathy; pAMR, pathologic antibody mediated rejection; PHM, predicted heart mass; PRA, panel reactive antibodies.

Characteristics	No. of patients (n = 544)	Study cohort
**Donor characteristics**		
Age, y, mean (SD)	520	32.8 (10.6)
Male, N (%)	519	428 (82.5)
**Recipient characteristics**		
Age, y, mean (SD)	544	53.9 (14.3)
Male, N (%)	544	436 (80.1)
**Race**		
Asian, N (%)	544	37 (6.8)
Black, N (%)	544	69 (12.7)
Native American, N (%)	544	4 (0.7)
Other Race, N (%)	544	35 (6.4)
Pacific Islander, N (%)	544	12 (2.2)
White, N (%)	544	387 (71.1)
**Ethnicity**		
Hispanic or Latino, N (%)	544	165 (30.3)
Recipient BMI, mean (SD)	521	26.5 (4.4)
**Indication for HTx**		
NICM, N (%)	544	325 (59.7)
ICM, N (%)	544	185 (34.0)
Congenital, N (%)	544	22 (4.0)
Cardiac allograft failure, N (%)	544	12 (2.2)

medRxiv preprint doi: https://doi.org/10.1101/2023.12.01.23299311; this version posted October 1, 2024. The copyright holder for this preprint (which was not certified by peer review) is the author/funder, who has granted medRxiv a license to display the preprint in perpetuity.
It is made available under a CC-BY 4.0 International license .

Allosensitization pre-HTx (PRA ≥ 10%), N (%)	457	85 (18.6)
Durable MCS, N (%)	543	188 (34.6)
**HTx characteristics**		
Multiorgan transplant, N (%)	544	76 (14.0)
Cold ischemic time, min, mean (SD)	520	200.3 (64.0)
Sex mismatch (female D-male R), N (%)	519	42 (8.1)
PHM difference, % recipient PHM, mean (SD)	513	5.2 (20.9)
Induction therapy, N (%)	519	256 (49.3)
DCD, N (%)	544	82 (15.1)
CMV mismatch (D+/R-), N (%)	531	105 (19.8)
**HTx outcomes**		
Primary graft dysfunction	542	118 (21.8)
History of pAMR positivity	544	75 (13.8)
pAMR1i	75	44 (58.7)
pAMR1h	75	5 (6.7)
pAMR2	75	26 (34.7)
Mixed ACR (ISHLT grade ≥ 2R) and pAMR	75	8 (10.7)
History of DSA positivity	544	140 (25.7)
De novo DSA	134	129 (96.3)
Class I de novo DSAs alone	129	23 (17.8)
Class II de novo DSAs alone	129	75 (58.1)
Both class I and II de novo DSAs	129	31 (24.0)

medRxiv preprint doi: https://doi.org/10.1101/2023.12.01.23299311; this version posted October 1, 2024. The copyright holder for this preprint (which was not certified by peer review) is the author/funder, who has granted medRxiv a license to display the preprint in perpetuity.
It is made available under a CC-BY 4.0 International license .

Concurrent cardiac allograft vasculopathy with initial pAMR positivity	20	5 (25.0)
Concurrent cardiac allograft dysfunction with initial pAMR positivity	72	17 (23.6)

medRxiv preprint doi: https://doi.org/10.1101/2023.12.01.23299311; this version posted October 1, 2024. The copyright holder for this preprint (which was not certified by peer review) is the author/funder, who has granted medRxiv a license to display the preprint in perpetuity.
It is made available under a CC-BY 4.0 International license .

Table S2. Causes of death compared across pAMR/DSA groups. DSA, donor-specific antibody; pAMR, pathologic antibody mediated rejection.

Outcomes	pAMR+/DSA+ (n = 45)	pAMR+/DSA- (n = 30)	pAMR-/DSA+ (n = 95)	pAMR-/DSA- (n = 374)	Total
All-cause mortality or cardiac retransplant (%)	13 (28.9)	4 (13.3)	6 (6.3)	38 (10.2)	61 (11.2)
Cardiovascular related death (%)	7 (15.6)	1 (3.3)	3 (3.2)	6 (1.6)	17 (3.1)
Infectious related mortality (%)	1 (2.2)	1 (3.3)	2 (2.1)	19 (5.1)	23 (4.2)
Cancer (%)	0	0	0	5 (1.3)	5 (0.9)
Other cause of death (%)	2 (4.4)	1 (3.3)	1 (1.1)	4 (1.1)	8 (1.5)
Unknown cause of death (%)	1 (2.2)	1 (3.3)	0	2 (0.5)	4 (0.7)

medRxiv preprint doi: https://doi.org/10.1101/2023.12.01.23299311; this version posted October 1, 2024. The copyright holder for this preprint (which was not certified by peer review) is the author/funder, who has granted medRxiv a license to display the preprint in perpetuity.
It is made available under a CC-BY 4.0 International license .

Table S3. Comparison of different de novo DSA patterns for diagnosis of pathologic antibody mediated rejection. DSA, donor-specific antibody. 95% confidence intervals are in parenthesis. #, reference is all other DSAs group.

De novo DSAs	Positive predictive value	p_c-value$^{\#}$	Odds ratio	p_c-value$^{\#}$
Both class I and II DSAs on initial DSA+ testing	64.2% (36.4%-88.9%)	0.031	6.44 (1.14-45.82)	0.016
Progression from one DSA to both class I and II DSAs	58.2% (28.6%-87.5%)	0.124	5.02 (0.81-35.88)	0.087
Two or more class II DSAs on initial DSA+ testing	52.9% (28.6%-77.3%)	0.103	4.05 (0.85-20.26)	0.087
All other DSAs	21.5% (12.9%-30.5%)	-	-	-

medRxiv preprint doi: https://doi.org/10.1101/2023.12.01.23299311; this version posted October 1, 2024. The copyright holder for this preprint (which was not certified by peer review) is the author/funder, who has granted medRxiv a license to display the preprint in perpetuity.
It is made available under a CC-BY 4.0 International license .

Table S4. Unipredictor and multipredictor Cox proportional hazards analyses for cardiac survival. Unipredictor parameters with a p-value $\leq$ 0.15 are included in addition to certain clinical parameters of interest. HRs and CIs are not provided for categorical variables in this table. ACR, acute cellular rejection; CI, confidence interval; cPRA, calculated panel reactive antibodies; DSA, donor-specific antibodies; ECMO, extracorporeal membrane oxygenation; HTx, heart transplantation; HR, hazard ratio; MCS, mechanical circulatory support; pAMR, pathologic antibody mediated rejection; PHM, predicted heart mass; pMCS, percutaneous mechanical circulatory support; UNOS, United Network for Organ Sharing. *, allosensitized patients defined as having a UNOS cPRA >= 10%.

**Predictors**	**Total # of patients (n = 544)**	**Total # of events (n = 21)**	**HR**	**95% CI**	**p-value**
**Unipredictor analysis**					
Recipient age (by 10-yr)	544	21	0.73	0.57-0.93	**0.010**
Recipient female sex (vs. recipient male sex)	544	21	1.48	0.57-3.82	0.423
Recipient race and ethnicity (vs. non-Hispanic White)	544	21	-	-	0.222
Multiorgan transplant (yes vs. no)	544	21	0.79	0.18-3.43	0.757
HTx indication (vs. non-ischemic cardiomyopathy)	544	21	-	-	0.661
Allosensitization pre-HTx*	457	16	1.19	0.38-3.68	0.769
Durable MCS at time of HTx (yes vs. no)	543	21	1.58	0.67-3.73	0.295
Medical nonadherence (yes vs. no)	544	21	4.15	1.75-9.82	**0.001**
Donor age (by 10-yr)	520	19	1.30	0.88-1.93	0.188
Induction therapy (yes vs. no)	519	19	1.22	0.48-3.13	0.675
Cold ischemic time (per hour)	519	19	0.84	0.53-1.33	0.454

medRxiv preprint doi: https://doi.org/10.1101/2023.12.01.23299311; this version posted October 1, 2024. The copyright holder for this preprint
(which was not certified by peer review) is the author/funder, who has granted medRxiv a license to display the preprint in perpetuity.
It is made available under a CC-BY 4.0 International license .

PHM difference (per % recipient PHM increment)	513	19	1.00	0.98-1.02	0.839
Donation after cardiac death (vs. brain death)	544	21	2.84	0.55-14.65	0.212
Primary graft dysfunction (yes vs. no)	542	21	3.31	1.36-8.08	**0.009**
ECMO pre-HTx (yes vs. no)	540	20	3.96	0.53-29.88	0.182
pMCS pre-HTx (yes vs. no)	540	20	0.28	0.04-2.10	0.215
De novo DSAs (vs. no DSA)	533	20	3.40	1.41-8.22	**0.007**
Class I de novo DSAs alone (vs. no DSA)	533	20	-	-	0.997
Class II de novo DSAs alone (vs. no DSA)	533	20	2.66	0.89-7.97	0.080
Both class I and II de novo DSAs (vs. no DSA)	533	20	7.16	2.54-20.14	**<0.001**
Sex mismatch (female D-male R vs. male D-male R)	519	19	1.71	0.49-5.93	0.397
pAMR/DSA group (vs. pAMR-/DSA- group)	544	21	-	-	**0.003**
Cardiac allograft vasculopathy (vs. CAV grades 0 or 1)	474	19	5.31	2.08-13.58	**<0.001**
Cardiac allograft dysfunction (yes vs. no)	544	21	6.30	2.65-14.98	**<0.001**
History of ACR > 1R (vs. ACR grades 0R/1R)	530	21	3.21	1.35-7.62	**0.008**
**Multipredictor analysis**					
Cardiac allograft	544	21	5.68	1.87-17.19	**0.002**

medRxiv preprint doi: https://doi.org/10.1101/2023.12.01.23299311; this version posted October 1, 2024. The copyright holder for this preprint (which was not certified by peer review) is the author/funder, who has granted medRxiv a license to display the preprint in perpetuity.
It is made available under a CC-BY 4.0 International license .

dysfunction					
Recipient Age	544	21	1.85	1.06-3.23	**0.029**
Donor Age	520	19	1.64	1.02-2.63	**0.040**
Medical nonadherence	544	21	2.62	0.92-7.48	0.071
Primary graft dysfunction	542	21	2.42	0.86-6.82	0.094
Cardiac allograft vasculopathy	474	19	2.57	0.83-7.94	0.100
