# Supplementary material for: Antibody Mediated Rejection is not Associated with Worse Survival in Adherent Heart Transplant Patients in the Contemporary Era
Source: medRxiv. 2023 Dec 8:2023.12.01.23299311. Preprint. [Version 2] doi: 10.1101/2023.12.01.23299311 (PMC10723500; doi:10.1101/2023.12.01.23299311)
Supplement: Supplementary file 1 [file NIHPP2023.12.01.23299311v2-supplement-1.pdf]

medRxiv preprint doi: https://doi.org/10.1101/2023.12.01.23299311; this version posted December 8, 2023. The copyright holder for this preprint (which was not certified by peer review) is the author/funder, who has granted medRxiv a license to display the preprint in perpetuity.
It is made available under a CC-BY 4.0 International license .



**Supplementary Figure S1.** Kaplan-Meier Curves for Freedom from C4d positivity in C4d+/DSA+ and C4d+/DSA- patients. Median time (diamond) to C4d positivity is 33.6 weeks from heart transplantation (IQR, 3.4-163.0 weeks) in C4d+/DSA+ patients. Median time (triangle) to C4d positivity is 3.6 weeks from heart transplantation (IQR, 1.6-9.4 weeks) in C4d+/DSA- patients. The C4d+/DSA+ group showed a significantly increased median time to C4d positivity compared to the C4d+/DSA- group (logrank p = 0.002).

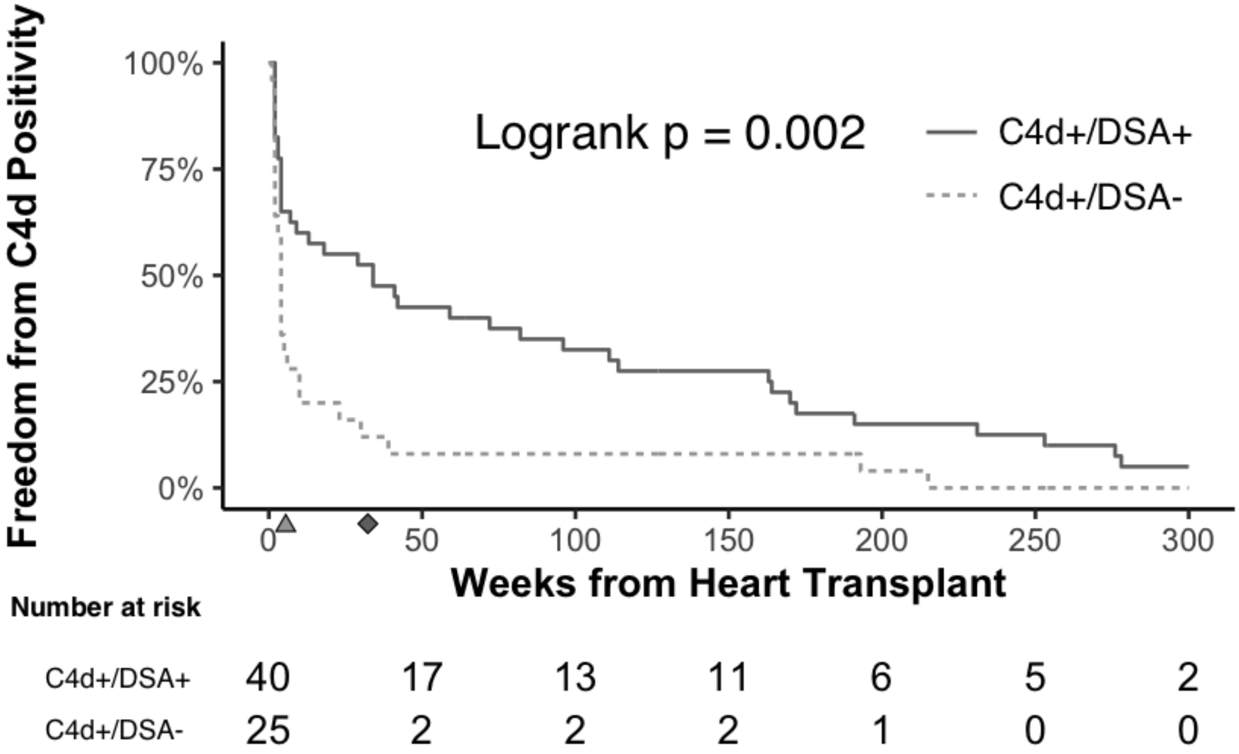

medRxiv preprint doi: https://doi.org/10.1101/2023.12.01.23299311; this version posted December 8, 2023. The copyright holder for this preprint (which was not certified by peer review) is the author/funder, who has granted medRxiv a license to display the preprint in perpetuity.
It is made available under a CC-BY 4.0 International license .



**Supplementary Table S1**. Patient outcomes compared across groups. ACR, acute cellular rejection; DSA, donor-specific antibody.

| | C4d+/DSA+ (n = 40) | C4d+/DSA- (n = 25) | C4d-/DSA+ (n = 94) | C4d-/DSA- (n = 360) |
|---|---|---|---|---|
| All-cause mortality or cardiac retransplant (%) | 11 (27.5) | 3 (12.0) | 6 (6.4) | 38 (10.6) |
| Cardiovascular related death (%) | 6 (15.0) | 1 (4.0) | 3 (3.2) | 6 (1.7) |
| Infectious related mortality (%) | 1 (2.5) | 0 | 2 (2.1) | 19 (5.3) |
| Cardiac allograft dysfunction (%) | 20 (50.0) | 3 (12.0) | 15 (16.0) | 43 (11.9) |
| Cardiac allograft vasculopathy (%) | 8 (24.2) | 2 (9.5) | 6 (8.0) | 14 (6.1) |
| Future ACR (%) | 9 (24.3) | 4 (16.0) | 12 (12.8) | 40 (11.2) |

medRxiv preprint doi: https://doi.org/10.1101/2023.12.01.23299311; this version posted December 8, 2023. The copyright holder for this preprint (which was not certified by peer review) is the author/funder, who has granted medRxiv a license to display the preprint in perpetuity.
It is made available under a CC-BY 4.0 International license .



**Supplementary Table S2**. Multipredictor model for all cause death or cardiac retransplant as the outcome. BMI, body mass index.

| Variables | HR | 95% CI | p-value |
|---|---|---|---|
| Cardiac allograft dysfunction | 2.89 | [1.38-6.04] | p = 0.005 |
| Medical nonadherence | 2.23 | [1.08-4.63] | p = 0.031 |
| Sex mismatch (female D-male R) | 2.59 | [1.09-6.15] | p = 0.031 |
| Recipient BMI (per kg/m$^2$) | 0.92 | [0.85-1.00] | p = 0.057 |
| Cardiac allograft vasculopathy | 2.10 | [0.84-5.20] | p = 0.111 |

medRxiv preprint doi: https://doi.org/10.1101/2023.12.01.23299311; this version posted December 8, 2023. The copyright holder for this preprint (which was not certified by peer review) is the author/funder, who has granted medRxiv a license to display the preprint in perpetuity.
It is made available under a CC-BY 4.0 International license .



**Supplementary Table S3**. Single predictor cox proportional hazards model for cardiac mortality or retransplant as the outcome. Hazard ratios and confidence intervals were not provided for categorical variables in this simplified table. AMR, antibody mediated rejection; BMI, body mass index; CMV, cytomegalovirus; cPRA, calculated panel reactive antibodies; DSA, donor-specific antibodies; ECMO, extracorporeal membrane oxygenation; HTx, heart transplantation; MCS, mechanical circulatory support; PHM, predicted heart mass; pMCS, percutaneous mechanical circulatory support. *, allosensitized patients defined as having a UNOS cPRA >= 10%.

| Variables | Total # of patients | Total # of events | Hazard ratio | 95% confidence interval | p-value |
|---|---|---|---|---|---|
| **Recipient characteristics** | | | | | |
| Age (per 1-y increment) | 519 | 20 | 0.96 | [0.94-0.99] | 0.004 |
| Female sex | 519 | 20 | 1.76 | [0.67-4.61] | 0.248 |
| Race and ethnicity | 519 | 20 | - | - | 0.219 |
| Recipient BMI (per $kg/m^2$) | 519 | 20 | 0.98 | [0.90-1.08] | 0.700 |
| Multi-organ transplant | 519 | 20 | 1.87 | [0.62-5.65] | 0.269 |
| HTx indication | 519 | 20 | - | - | 0.580 |
| Allosensitization pre-HTx* | 449 | 15 | 1.27 | [0.40-3.99] | 0.685 |
| Blood type | 512 | 20 | - | - | 0.553 |
| Durable MCS at time of HTx | 519 | 20 | 1.32 | [0.55-3.18] | 0.537 |
| Medical nonadherence | 519 | 20 | 4.52 | [1.87-10.94] | 0.001 |
| **Donor characteristics** | | | | | |
| Age (per 1-y increment) | 507 | 18 | 1.02 | [0.98-1.06] | 0.387 |
| Donor BMI (per $kg/m^2$) | 501 | 18 | 1.01 | [0.94-1.09] | 0.809 |
| **Transplant characteristics** | | | | | |
| Induction therapy | 505 | 18 | 1.12 | [0.43-2.92] | 0.816 |

medRxiv preprint doi: https://doi.org/10.1101/2023.12.01.23299311; this version posted December 8, 2023. The copyright holder for this preprint (which was not certified by peer review) is the author/funder, who has granted medRxiv a license to display the preprint in perpetuity.
It is made available under a CC-BY 4.0 International license .



| | | | | | |
|---|---|---|---|---|---|
| Cold ischemic time (per min) | 505 | 18 | 1.00 | [0.99-1.01] | 0.457 |
| PHM difference (per % recipient PHM increment) | 501 | 18 | 1.01 | [0.99-1.03] | 0.486 |
| Donation after cardiac death | 519 | 20 | 3.43 | [0.63-18.63] | 0.153 |
| ECMO pre-HTx | 515 | 19 | 3.99 | [0.53-30.23] | 0.180 |
| ECMO post-HTx | 515 | 19 | 4.48 | [1.02-19.63] | 0.047 |
| pMCS pre-HTx | 515 | 19 | 0.29 | [0.04-2.19] | 0.230 |
| pMCS post-HTx | 515 | 19 | 2.26 | [0.74-6.91] | 0.153 |
| History of C4d positivity | 519 | 20 | 3.96 | [1.63-9.60] | 0.002 |
| Number of AMR episodes (per episode) | 518 | 19 | 2.07 | [1.33-3.23] | 0.001 |
| History of DSA positivity | 519 | 20 | 2.86 | [1.18-6.91] | 0.020 |
| Class 1 Antibodies | 519 | 20 | 3.86 | [1.54-9.70] | 0.004 |
| Class 2 Antibodies | 519 | 20 | 3.72 | [1.54-8.98] | 0.004 |
| CMV mismatch | 511 | 19 | - | - | 0.471 |
| Sex mismatch | 506 | 18 | - | - | 0.648 |
| C4d/DSA Group | 519 | 20 | - | - | 0.011 |
| Cardiac allograft vasculopathy | 360 | 18 | 5.20 | [2.00-13.50] | 0.001 |
| Cardiac allograft dysfunction | 518 | 20 | 5.66 | [2.34-13.69] | <0.001 |

medRxiv preprint doi: https://doi.org/10.1101/2023.12.01.23299311; this version posted December 8, 2023. The copyright holder for this preprint (which was not certified by peer review) is the author/funder, who has granted medRxiv a license to display the preprint in perpetuity.
It is made available under a CC-BY 4.0 International license .



**Supplementary Table S4**. Multipredictor analysis for donor-specific antibody (DSA) positivity as the outcome. cPRA, calculated panel of reactive antibodies; HTx, heart transplantation; MCS, mechanical circulatory support; UNOS, United Network for Organ Sharing. *, allosensitized patients defined as having a UNOS cPRA >= 10%.

| Variables | HR | 95% CI | p-value |
|---|---|---|---|
| Recipient Age (per 1-y increment) | 0.98 | [0.96-0.99] | p < 0.001 |
| Medical nonadherence | 2.03 | [1.33-3.08] | p = 0.001 |
| Allosensitization pre-HTx* | 1.55 | [1.02-2.36] | p = 0.039 |
| Durable MCS at time of HTx | 0.71 | [0.48-1.05] | p = 0.083 |

medRxiv preprint doi: https://doi.org/10.1101/2023.12.01.23299311; this version posted December 8, 2023. The copyright holder for this preprint (which was not certified by peer review) is the author/funder, who has granted medRxiv a license to display the preprint in perpetuity.
It is made available under a CC-BY 4.0 International license .



**Supplementary Table S5**. Multipredictor model for C4d positivity as the outcome. BMI, body mass index.

| Variables | HR | 95% CI | p-value |
|---|---|---|---|
| Class 1 antibodies | 3.04 | [1.73-5.35] | $p < 0.001$ |
| Class 2 antibodies | 2.97 | [1.71-5.16] | $p < 0.001$ |
| Medical nonadherence | 1.89 | [1.11-3.21] | $p = 0.019$ |
| Female recipient sex | 1.77 | [1.03-3.05] | $p = 0.038$ |
| Recipient BMI (per kg/m$^2$) | 0.96 | [0.92-1.01] | $p = 0.126$ |

medRxiv preprint doi: https://doi.org/10.1101/2023.12.01.23299311; this version posted December 8, 2023. The copyright holder for this preprint (which was not certified by peer review) is the author/funder, who has granted medRxiv a license to display the preprint in perpetuity.
It is made available under a CC-BY 4.0 International license .



**Supplementary Table S6**. Single predictor cox proportional hazards model for cardiac allograft dysfunction as the outcome. Hazard ratios and confidence intervals were not provided for categorical variables in this simplified table. AMR, antibody mediated rejection; BMI, body mass index; CMV, cytomegalovirus; cPRA, calculated panel reactive antibodies; DSA, donor-specific antibodies; ECMO, extracorporeal membrane oxygenation; HTx, heart transplantation; MCS, mechanical circulatory support; PHM, predicted heart mass; pMCS, percutaneous mechanical circulatory support. *, allosensitized patients defined as having a UNOS cPRA >= 10%.

| Variables | Total # of patients | Total # of events | Hazard ratio | 95% confidence interval | p-value |
|---|---|---|---|---|---|
| **Recipient characteristics** | | | | | |
| Age (per 1-y increment) | 519 | 78 | 1.01 | [0.99-1.02] | 0.568 |
| Female sex | 519 | 78 | 0.84 | [0.49-1.45] | 0.538 |
| Race and ethnicity | 519 | 78 | - | - | 0.593 |
| Recipient BMI (per kg/m$^2$) | 519 | 78 | 0.97 | [0.92-1.02] | 0.197 |
| Multiorgan transplant | 519 | 78 | 1.16 | [0.63-2.15] | 0.631 |
| HTx indication | 519 | 78 | - | - | 0.026 |
| Allosensitization pre-HTx* | 449 | 66 | 0.87 | [0.47-1.64] | 0.671 |
| Blood type | 512 | 77 | - | - | 0.664 |
| Durable MCS at time of HTx | 519 | 78 | 1.36 | [0.87-2.13] | 0.180 |
| Medical nonadherence | 519 | 78 | 2.49 | [1.56-3.97] | <0.001 |
| **Donor characteristics** | | | | | |
| Age (per 1-y increment) | 507 | 76 | 0.99 | [0.97-1.01] | 0.256 |
| Donor BMI (per kg/m$^2$) | 501 | 73 | 0.97 | [0.93-1.01] | 0.140 |
| **Transplant characteristics** | | | | | |
| Induction therapy | 505 | 75 | 0.79 | [0.50-1.26] | 0.319 |

medRxiv preprint doi: https://doi.org/10.1101/2023.12.01.23299311; this version posted December 8, 2023. The copyright holder for this preprint (which was not certified by peer review) is the author/funder, who has granted medRxiv a license to display the preprint in perpetuity.
It is made available under a CC-BY 4.0 International license .



| | | | | | |
|---|---|---|---|---|---|
| Cold ischemic time (per min) | 505 | 76 | 1.00 | [0.99-1.00] | 0.375 |
| PHM difference (per % recipient PHM increment) | 501 | 73 | 1.01 | [1.00-1.02] | 0.011 |
| Donation after cardiac death | 519 | 78 | 1.41 | [0.71-2.81] | 0.326 |
| ECMO pre-HTx | 515 | 77 | 2.64 | [0.83-8.41] | 0.100 |
| ECMO post-HTx | 515 | 77 | 2.76 | [1.19-6.37] | 0.018 |
| pMCS pre-HTx | 515 | 77 | 0.57 | [0.28-1.15] | 0.116 |
| pMCS post-HTx | 515 | 77 | 1.43 | [0.79-2.61] | 0.240 |
| History of C4d positivity | 519 | 78 | 2.45 | [1.50-3.98] | <0.001 |
| Number of AMR episodes (per episode) | 518 | 77 | 1.51 | [1.12-2.03] | 0.007 |
| History of DSA positivity | 519 | 78 | 2.07 | [1.32-3.23] | 0.001 |
| Class 1 Antibodies | 519 | 78 | 3.13 | [1.90-5.17] | <0.001 |
| Class 2 Antibodies | 519 | 78 | 2.48 | [1.58-3.89] | <0.001 |
| CMV mismatch | 511 | 78 | - | - | 0.365 |
| Sex mismatch | 506 | 76 | - | - | 0.312 |
| C4d/DSA Group | 519 | 76 | - | - | <0.001 |
| Cardiac allograft vasculopathy | 360 | 62 | 2.27 | [1.21-4.28] | 0.011 |

medRxiv preprint doi: https://doi.org/10.1101/2023.12.01.23299311; this version posted December 8, 2023. The copyright holder for this preprint (which was not certified by peer review) is the author/funder, who has granted medRxiv a license to display the preprint in perpetuity.
It is made available under a CC-BY 4.0 International license .



**Supplementary Table S7**. Pairwise comparisons of C4d/DSA groups for cardiac allograft dysfunction as the outcome. DSA, donor-specific antibodies. #, reference is C4d-/DSA- group.

| Variables | HR | 95% CI | p-value |
|---|---|---|---|
| C4d+/DSA+# | 3.16 | [1.51-6.61] | $p_c$ = 0.013 |
| C4d+/DSA-# | 0.63 | [0.15-2.65] | $p_c$ = 1.000 |
| C4d-/DSA+# | 1.26 | [0.63-2.49] | $p_c$ = 1.000 |

medRxiv preprint doi: https://doi.org/10.1101/2023.12.01.23299311; this version posted December 8, 2023. The copyright holder for this preprint (which was not certified by peer review) is the author/funder, who has granted medRxiv a license to display the preprint in perpetuity.
It is made available under a CC-BY 4.0 International license .



**Supplementary Table S8**. Pairwise comparisons of C4d/DSA groups for cardiac allograft vasculopathy as the outcome. Pairwise comparisons were adjusted for the identified confounders of donor age, cold ischemic time, and predicted heart mass difference by the forward model selection procedure. DSA, donor-specific antibodies. #, reference is C4d-/DSA- group.

| Variables | HR | 95% CI | p-value |
|---|---|---|---|
| C4d+/DSA+# | 3.52 | [1.21-10.24] | $p_c = 0.063$ |
| C4d+/DSA-# | 1.63 | [0.36-7.34] | $p_c = 0.527$ |
| C4d-/DSA+# | 1.98 | [0.72-5.43] | $p_c = 0.370$ |
